# Process Optimization of Scaled-Up Production and Biosafety Evaluation of the Dimethyl-Dioctadecyl-Ammonium Bromide/Poly(lactic acid) Nano-Vaccine

**DOI:** 10.3390/jfb15050127

**Published:** 2024-05-14

**Authors:** Hengye Yang, Yuan Gao, Meijuan Liu, Juan Ma, Qun Lu

**Affiliations:** 1 Beijing Chao-Yang Hospital, Capital Medical University, Beijing 100020, China; 2011019@mail.ccmu.edu.cn; 2 Key Laboratory of Forest Plant Ecology, Ministry of Education, Heilongjiang Provincial Key Laboratory of Ecological Utilization of Forestry-Based Active Substances, College of Chemistry, Chemistry Engineering and Resource Utilization, Northeast Forestry University, Harbin 150040, China; m18342201571@163.com; 3 College of Pharmacy, Heilongjiang University of Chinese Medicine, Harbin 150040, China; lmj42877@163.com; 4 Beijing Shijitan Hospital, Capital Medical University, Beijing 100038, China

**Keywords:** poly(lactic acid), scale preparation, nano-vaccine

## Abstract

Nano-adjuvant vaccines could induce immune responses and enhance immunogenicity. However, the application and manufacturing of nano-adjuvant is hampered by its challenging scale-up, poor reproducibility, and low security. Therefore, the present study aimed to optimize the preparation nanoparticles (NPs) using FDA-approved biopolymer materials poly(lactic acid) (PLA) and cationic lipid didodecyl-dimethyl-ammonium bromide (DDAB), develop the scale-up process, and evaluate the stability and biosafety of it. The optimum preparation conditions of DDAB/PLA NPs on a small scale were as follows: DDAB amount of 30 mg, aqueous phase volume of 90 mL, stirring rate at 550 rpm, and solidifying time of 12 h. Under the optimum conditions, the size of the NPs was about 170 nm. In scale-up preparation experiments, the vacuum rotary evaporation of 6 h and the Tangential flow ultrafiltration (TFU) method were the optimum conditions. The results suggested that DDAB/PLA NPs exhibited a uniform particle size distribution, with an average size of 150.3 ± 10.4 nm and a narrow polydispersity index (PDI) of 0.090 ± 0.13, coupled with a high antigen loading capacity of 85.4 ± 4.0%. In addition, the DDAB/PLA NPs can be stored stably for 30 days and do not have side effects caused by residual solvents. For biosafety, the acute toxicity experiments showed good tolerance of the vaccine formulation even at a high adjuvant dose. The local irritation experiment demonstrated the reversibility of muscular irritation, and the repeated toxicity experiment revealed no significant necrosis or severe lesions in mice injected with the high-dose vaccine formulation. Overall, the DDAB/PLA NPs exhibit potential for clinical translation as a safe candidate vaccine adjuvant.

## 1. Introduction

Vaccines constitute a pivotal defense against infectious diseases. Nevertheless, without adjuvants, subunit and genetically engineered vaccines often exhibit limited immunogenicity [1]. Since the 1930s, aluminum adjuvants have dominated the adjuvant landscape as the Food and Drug Administration (FDA)-approved adjuvants for human vaccines [2]. These adjuvants function by entrapping antigens at the injection site, forming a depot that gradually releases the antigens and stimulates a protective immune response [3,4]. In addition, aluminum adjuvants have been prized for embodying the ideal adjuvant characteristics, such as cost-effectiveness, safety, and ease of synthesis. Yet, with advancing clinical trial data on pharmacokinetics and toxicological risks, scientists are voicing concerns over the safety profile of aluminum adjuvants [5,6,7]. Notably, aluminum-adjuvanted vaccines have been implicated in pathological immune responses, potentially triggering autoimmune/inflammatory syndrome (ASIA syndrome), macrophagic myofasciitis (MMF), Gulf War Syndrome (GWS), tissue damage, and cytokine storms [8,9]. Moreover, the limitations of aluminum adjuvants in efficiently stimulating cell-mediated immunity, including cytotoxic T lymphocyte activity, have rendered them increasingly insufficient in advanced modern vaccine formulations [10,11]. Given these concerns, developing novel adjuvants is imperative.

In recent years, biodegradable polymer nanoparticles (NPs) have emerged as a new adjuvant and have received extensive attention from researchers due to their ability to encapsulate and deliver a variety of therapeutic agents, including drugs, proteins, and genes from degradation and premature release, improving its stability and bioavailability [12,13,14]. Poly(lactic acid) (PLA) is one of the widely used biodegradable polymers in vaccine delivery, and it was approved for human use by the FDA in 2004 [15]. PLA NPs have the advantages of non-toxicity, controllable particle size, and the ability to maximize the body’s immune response [16,17]. However, the previous use of the nanoprecipitation method for the preparation of didodecyl-dimethyl-ammonium bromide (DDAB)/PLA NPs is still at the laboratory scale [18,19], which is challenging to meet the needs of product development. Furthermore, introducing a vaccine formulation into the body can trigger excessive immune responses that lead to serious health issues, such as physiological dysfunction and tissue damage [20]. In extreme cases, this can result in life-threatening cytokine storms. Hence, a crucial step in vaccine development is an animal preclinical safety assessment. This aims to predict the safety of the vaccine’s clinical use in humans, minimize the risk of adverse effects in clinical trial participants, and inform vaccination protocols. However, the safety of DDAB/PLA NP vaccine preparations has not been systematically evaluated. Moreover, the measurement of residual organic solvents in the NPs is a parameter of utmost importance in green chemistry and pharmaceutical manufacturing. Therefore, if DDAB/PLA NPs are to be further applied and manufactured, it is necessary to develop a scale-up production process to obtain repeatable, stable, environmentally friendly, and safe NPs.

This study aimed to develop a new preparation technology for DDAB/PLA NPs on a laboratory scale using the nanoprecipitation method and optimize process formulations to achieve scale-up production. In addition, the stability and the organic solvent residues in DDAB/PLA NPs were also measured. Furthermore, the biosafety evaluation of the acute toxicity, repeated dose toxicity, and local muscle irritation experiments were systematically tested here to confirm the vaccine’s safety. This study provides a reference for the transformation of nanoparticle adjuvant vaccines from laboratory-scale to large-scale preparation.

## 2. Materials and Methods

### 2.1. Materials and Animals

PLA (molecular weight [MW]: 10 kDa) was purchased from Jinan Daigang Biomaterial Co., Ltd. (Jinan, China). DDAB was obtained from Sigma-Aldrich Chemical Co. (St. Louis, MO, USA). Beijing Chemical Works (Beijing, China) provided anhydrous ethanol (≥99.7%) and acetone (≥99.5%). OVA was purchased from Sigma-Aldrich Chemical Co. (St. Louis, MO, USA). Micro-BCA assay kits were bought from Thermo Fisher Scientific Inc. (Waltham, MA, USA). All other chemical reagents were of analytical grade. BALB/c mice (4–6 weeks and 6–8 weeks) under the SPF condition were provided by Vital River Laboratories (Beijing, China). 

### 2.2. Preparation of DDAB/PLA NPs

**Figure 1:**
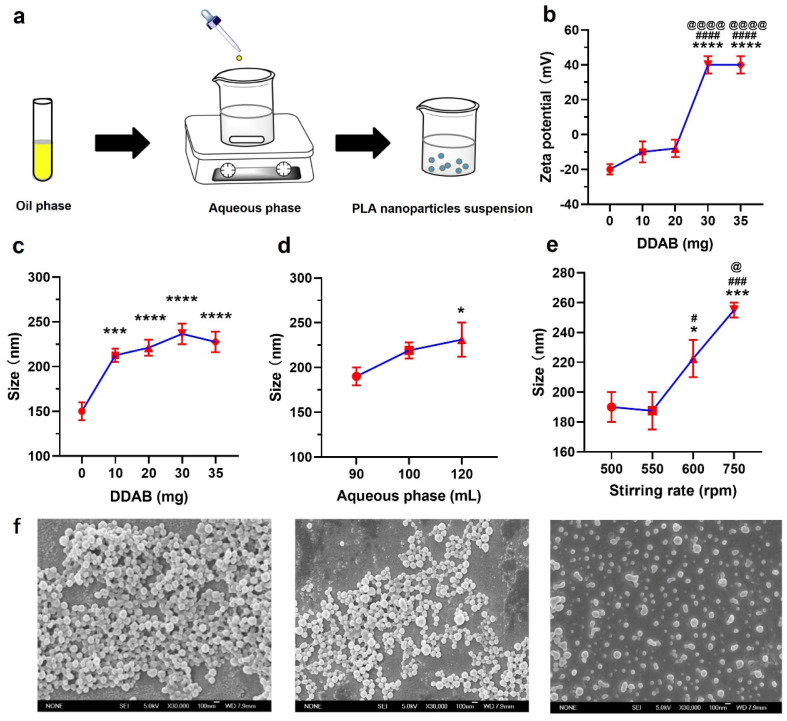
Optimization of the preparation of dimethyl-dioctadecyl-ammonium bromide (DDAB)/poly(lactic acid) (PLA) nanoparticles (NPs). (**a**) Schematic diagram of the preparation of DDAB/PLA NPs by nanoprecipitation, (**b**,**c**) effect of different the amount of DDAB on zeta potential and size of NPs, *** *p* < 0.001 and **** *p* < 0.0001 compared to 0 mg of DDAB; #### *p* < 0.0001 compared to 10 mg of DDAB; @@@@ *p* < 0.0001 compared to 20 mg of DDAB, (**d**) effect of different the volume of aqueous phase on the size of NPs, * *p* < 0.05 compared to 90 mL of the volume of aqueous phase, (**e**) effect of different stirring rate on size of NPs, * *p* < 0.05 and *** *p* < 0.001 compared to 500 rpm; # *p* < 0.05 and ### *p* < 0.001 compared to 550 rpm; @ *p* < 0.05 compared to 600 rpm, and (**f**) scanning electron microscope (SEM) images of DDAB/PLA NPs were solidified for 12 h, 24 h, and 36 h, respectively.

For the nanoprecipitation method (Figure 1a), 30 mg of DDAB and 100 mg of PLA were dissolved in a 15 mL mixture solvent of anhydrous ethanol and acetone (1:1, *v*/*v*) as the oil phase. As an aqueous phase, 90 mL of deionized water was added to a 250 mL beaker. Then, the oil phase was dropwise to the aqueous phase with a magnetic stirring (550 rpm). The organic solvent was removed by evaporating nanodroplets under a magnetic stirrer stirring at room temperature for a certain time at a specified speed. After that, the NPs were washed three times by centrifuging at 20,000× *g* for 15 min to remove the supernatant. The DDAB/PLA NPs were collected after centrifuging (5000× *g*, 10 min) to remove larger-sized particles.

**Figure 2:**
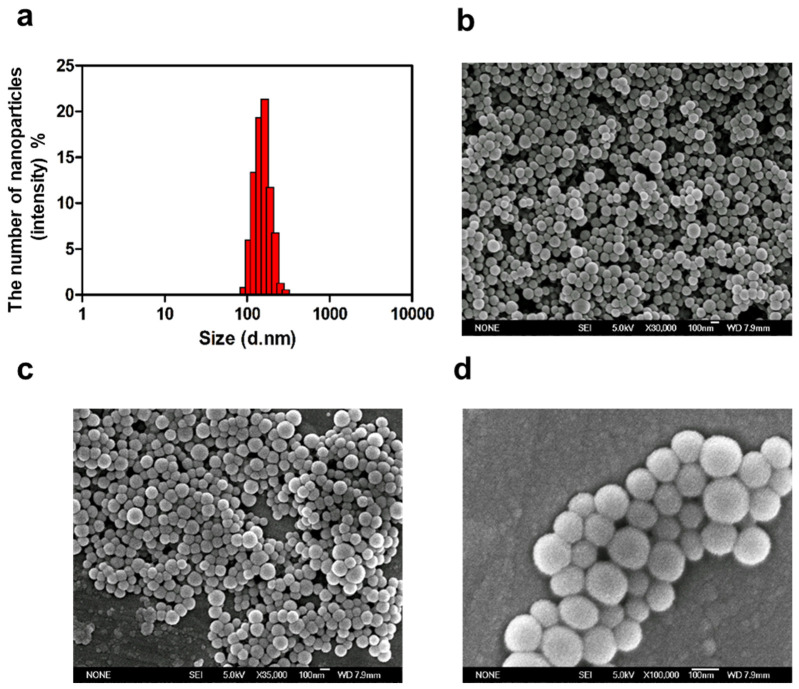
Characterization of DDAB/PLA NPs under optimal conditions. (**a**) Size distribution of DDAB/PLA NPs, (**b**–**d**) and SEM images of DDAB/PLA NPs at different magnifications ((**b**): ×30,000; (**c**): ×35,000; (**d**): ×100,000).

For the vacuum evaporation method, the preparation process was magnified ten times (Figure 2a). Briefly, DDAB (0.3 g) and PLA (1.0 g) were solubilized in a 150 mL mixture solvent of anhydrous ethanol and acetone (1:1, *v*/*v*) as the oil phase and 900 mL of deionized water was added into a reaction vessel as an aqueous phase. Then, the oil phase was gradually mixed with the deionized water phase under constant rotational speed. After adding, the mixture was transferred to a 2 L flask, and vacuum-assisted rotary evaporation was performed to remove the organic solvent. In addition to centrifugal washing, tangential flow filtration using a vivaflow50 membrane was also applied in the process of wash and concentration. 

### 2.3. Characterization of DDAB/PLA NPs

The size, polydispersity index (PDI), and zeta potential were characterized by a laser particle size analyzer (Nano ZS, Malvern Instruments, Malvern, UK). In total, 1 mL of NPs (2 mg/mL) were added into a quartz cuvette to detect the particle size and PDI. Then, the NPs were transferred into a potential sample cell that examined the zeta potential. A field emission scanning electron microscope (FE-SEM) (JSM-6700F, Jeol, Tokyo, Japan) was used to observe the morphology of NPs. The NPs were dropped onto a flat aluminum foil surface, dried at room temperature, sputter-coated with gold-palladium (20 mA, 120 s), and then scanned at the acceleration voltage of 5.0 kV. The antigen adsorption efficiency was determined by the decrement method using the MicroBCA assay kit [21]. In total, 250 μg/mL of OVA antigen was adsorbed with 3 mg/mL of NPs at room temperature for 4 h. After centrifugation with a 100 KD ultrafiltration tube, OVA concentration in the supernatant was detected, and the antigen adsorption efficiency was calculated using the following formula:

(1) $$Antigen\;adsorption\;efficiency\;\left(\%\right)=\frac{Total\;of\;OVA-OVA\;in\;supernatant}{Total\;of\;OVA}\times 100$$

### 2.4. Evaluation of Formulation Stability

The DDAB/PLA NPs were prepared using vacuum evaporation and washed using tangential flow filtration. Then, the NPs were stored at room temperature and 4 °C until 30 d, respectively. The size, zeta potential, antigen adsorption efficiency, and morphology of NPs were characterized at 0, 5, 10, 15, 21, and 30 d.

### 2.5. Detection of the Residual Organic Solvents in the NPs

The residual organic solvents of ethanol and acetone in the 10 mg/mL prepared DDAB/PLA NPs were analyzed using gas chromatography (GC). The chromatographic column of DB-624 (30 m × 0.530 mm × 3.00 µm) was used to analyze, obtained from Agilent Technologies Inc. (Santa Clara, CA, USA). The detector was a hydrogen flame ionization detector (FID), and nitrogen was the carrier gas. The inlet temperature was 180 °C, divided into 66:10:1, the detector temperature was 230 °C, and the initial column temperature was 80 °C. The programmed heating method: equilibrium at 80 °C for 5 min, 30 °C per min up to 180 °C, and then equilibrium at 180 °C for 1 min.

### 2.6. Acute Toxicity Experiment

BALB/c mice (4–6 weeks) were randomly assigned into five groups, with three females and three males in each group. A total of 100 μL of DDAB/PLA NPs dissolved in 0.9% saline were injected intramuscularly into the right hind leg of mice at dosages of 0.3, 3, 15, and 30 mg/mouse, and the OVA was administered at a dose of 25 μg/mouse. The PBS was the blank control. Before administration, the mice were weighed, and their weight was recorded at fixed intervals every day after administration until 14 d. In addition, the general behavior of the mice was observed continuously for 1 h immediately after injections and then intermittently observed for 24 h. All mice were further observed for up to 14 days after treatment, and their general physical states, including eating and movement, were monitored. In the event of animal death during the experiment, gross anatomy should be performed and analyzed in time. The toxic effects were evaluated based on the maximum tolerated dose (MTD), which was determined to be the highest administered dose and did not result in animal deaths. The MTD was considered greater than the dose administered if no animal deaths occurred.

### 2.7. Comparison of Local Irritation Reactions

To evaluate local irritation of the DDAB/PLA NPs, we randomly divided purchased BALB/c mice (female, 6–8 weeks) into 2 groups with 4 mice per group. The dose of 0.3 mg/mouse DDAB/PLA NPs and PBS (blank control) was administered intramuscularly each to the right hind leg of mice, respectively. After administration, the mice’s activity, food intake, and water consumption were observed daily. The mice were killed, and the irritation reaction of the injected muscle site was observed day 2 and day 14 after injection, respectively. In addition, the injected muscle site was fixed in 4% paraformaldehyde, embedded into paraffin, and cut to yield 4-mm thick sections and further was evaluated by hematoxylin and eosin (H&E) stained tissue sections using an automatic quantitative pathological analysis system Perkin Elmer, Inc. (Waltham, MA, USA). 

### 2.8. Repeated Toxicity Experiment 

60 BALB/c mice (4–6 weeks) were divided into 5 groups, with six male and six female mice in each group. The mice received intramuscular injections in the right hind leg muscle at immunization time on day 0, day 14, and day 28. In addition, before each injection and 4 h after injection, the rectal temperature of the mice was measured using a mouse rectal thermometer probe (Shenzhen Shanya Instrument Co., Ltd., Shenzhen, China) after lubricating with white petrolatum. Fourteen days after administration, all mice were euthanized, and the major organs, lymph nodes, and thymus were collected. Then, they were fixed in a 10% paraformaldehyde solution overnight and underwent H&E-stained processing. The weights of significant organs were determined, and the organ coefficients were calculated using the following formula:

(2) $$Organ\;Coefficient\;\left(\frac{mg}{g}\right)=\frac{Organ\;weight\left(mg\right)}{Mouse\;body\;weight\;\left(g\right)}\;$$

### 2.9. Statistical Analysis

The experimental data were analyzed and statistically evaluated using GraphPad Prism 5 software. The results were presented as mean ± standard deviation. The one-way ANOVA-Tukey’s multiple test method was employed for comparing the differences among the groups, and *p* < 0.05 was considered that there was a significant difference between the two groups.

## 3. Results and Discussion

### 3.1. Optimization of the Preparation of DDAB/PLA NPs

Before the large-scale preparation of the DDAB/PLA nano-vaccine, the optimal experimental conditions for preparing DDAB/PLA NPs were first explored under laboratory-scale conditions. The process of preparing DDAB/PLA NPs using nanoprecipitation is shown in Figure 1a. Considering the key influencing factors in the preparation process, four factors (DDAB amounts, volume of the aqueous phase, stirring rate, and solidifying time) were optimized.

#### 3.1.1. The Effect of DDAB Amounts on Physicochemical Characteristics of the Prepared NPs

PLA NPs with negative surface charge were reversed to positive by cationic lipid -DDAB to adsorb antigens with negative potential and improve their stability [3]. To determine the appropriate amount of DDAB to add, different amounts of DDAB between 0 and 35 mg were optimized. According to the results in Figure 1b,c, the zeta potential of the particles also increased with the enhancement of DDAB amounts, while their size increased until the DDAB amounts reached 30 mg. The PLA NPs are slightly aggregated after being solidified overnight when the DDAB amount is 0 mg. This may be because the PLA NPs had a negative charge without DDAB, so the Van der Waals force between the NPs was more significant than the repulsive force. In addition, the NPs flocculated directly after overnight when the amounts of DDAB were 10 mg and 20 mg, which may be due to the insufficient addition of DDAB as a cationic lipid and surfactant, which could not form stable DDAB/PLA NPs. Considering the toxicity of DDAB, 30 mg of DDAB was selected for further optimal experiments.

#### 3.1.2. The Effect of the Volume of the Aqueous Phase on Physicochemical Characteristics of the Prepared NPs

In this study, the effect of different volumes of the aqueous phase (90 to 120 mL) on the particle size of NPs was investigated. It has been observed that the volume of the aqueous phase can significantly affect the particle size of the resulting NPs (Figure 1d). With an increase in the aqueous phase volume, the size of NPs was gradually enlarged. The rise of water phase volume probably caused this, leading to insufficient stirring and poor sphericity when the rotation speed is constant. It can be noticed that the volume of the aqueous phase was 90 mL, and the particle size was below 200 nm. Studies have shown that the size of NPs in the range of 20–200 nm not only safeguards the antigens from degradation but also enhances their presentation and facilitates absorption by professional antigen-presenting cells. As a result, these NPs enable better recognition and uptake by bone marrow dendritic cells (BMDCs) compared to the antigen alone [22,23]. Therefore, the aqueous phase volume of 90 mL was chosen in the further optimization experiments.

#### 3.1.3. The Effect of Stirring Rate on Physicochemical Characteristics of the Prepared NPs

The fluid shear force in the solution and volatilization of the organic solvent is determined by the stirring rate in the preparation process, which plays a central role in the morphology of polymer NPs [24]. According to the results of the preliminary experiments, the DDAB/PLA NPs exhibited a spherical shape, and their particle size was about 200 nm when prepared under the conditions of 500–750 rpm. From Figure 1e, the particle size of the NPs gradually increased with the increase in stirring rate. In addition, the particle size was larger than 200 nm under 600 and 750 rpm conditions. The reason may be that the NPs will aggregate under dramatic stirring, increasing the particle size. Therefore, to obtain NPs with a smaller particle size, it is necessary to control the stirring rate at 550 rpm. 

#### 3.1.4. The Effect of Solidifying Time on Physicochemical Characteristics of the Prepared NPs

The solidifying time is an important parameter that can influence the surface morphology of nanoparticles. However, the effects of different solidification times on the surface morphology of nanoparticles have not been fully understood. Therefore, this study aimed to investigate the impact of solidifying time on the surface morphology of nanoparticles. A controlled solidifying process was used to solidify suspensions of nanoparticles for 12, 24, and 36 h. The surface morphology of the resulting nanoparticles was characterized using SEM (Figure 1f). The results show that prolonging the solidifying time did not significantly improve the sphericity, especially since 36 h of the solidifying time will obtain fewer NPs. Therefore, the effect of solidifying time on the properties of NPs is not as good as that of other factors. To save time, 12 h was selected as the best condition.

### 3.2. Characterization of DDAB/PLA NPs under the Optimal Conditions

To confirm the stability and reliability of the optimal conditions, the DDAB/PLA NPs were prepared three times under the optimal conditions (the DDAB dosage was 30 mg, the volume of the aqueous phase was 90 mL, the stirring rate was 550 rpm, and the solidifying time was 12 h) and the results are shown in Figure 2. The particle size of the prepared DDAB/PLA NPs was about 170 nm with a relatively uniform particle size distribution (Figure 2a). The SEM image showed that the surface of the NPs is smooth and regular, even at ×100,000 magnification, no adhesion was observed under observation (Figure 2b–d). Therefore, the results indicated that the DDAB/PLA NPs prepared under optimal conditions have a relatively uniform size distribution and smooth surface morphology. The stability and reliability of the optimal conditions were confirmed through repeated preparations, indicating that these conditions can be used to prepare DDAB/PLA NPs with desired properties reproducibly.

### 3.3. Optimization of the Scale-Up Process of DDAB/PLA NPs

#### 3.3.1. The Effect of the Time of Removal of Organic Solvents by Vacuum Rotary Evaporation on Physicochemical Characteristics of the Prepared NPs

**Figure 3:**
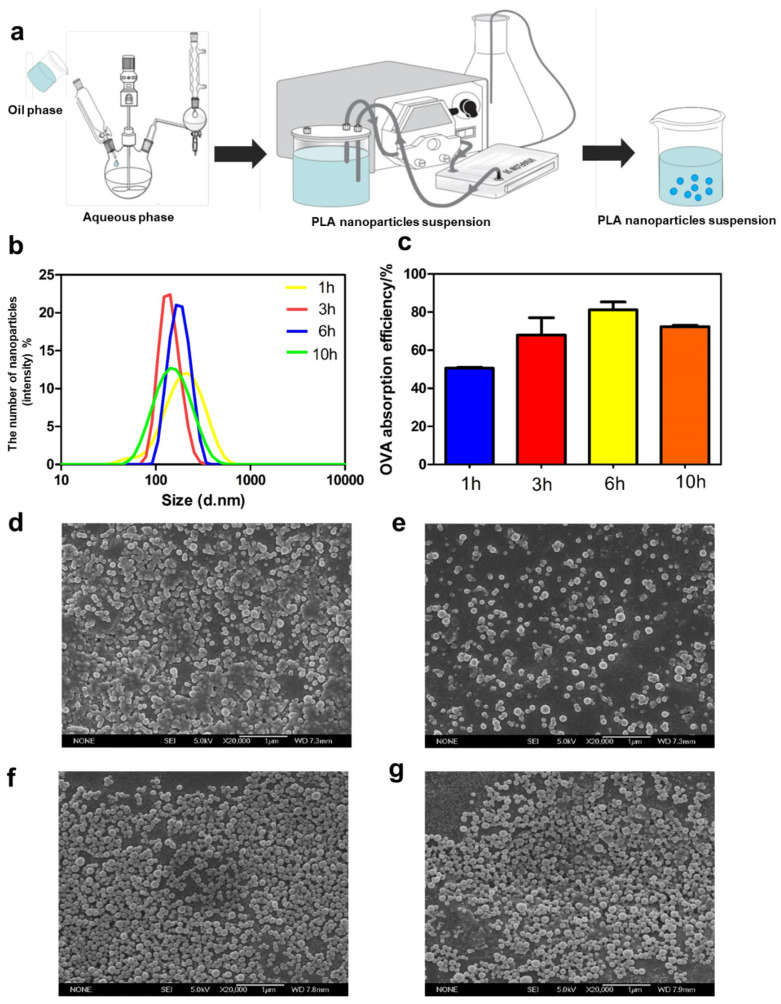
Effect of different vacuum rotary evaporation time on DDAB/PLA NPs. (**a**): Schematic diagram of the scale-up process of DDAB/PLA NPs; (**b**) size distributions of DDAB/PLA NPs; (**c**) antigen loading efficiencies; (**d**–**g**) SEM images of DDAB/PLA NPs (×20,000) under different vacuum rotary evaporation time ((**d**), 1 h; (**e**), 3 h; (**f**), 6 h; (**g**), 10 h).

The traditional nanoprecipitation method requires over 12 h of evaporation to remove organic reagents using magnetic stirring, significantly decreasing the preparation efficiency. A vacuum rotary evaporation method was employed for scale-up preparing NPs, which showed the benefits of high productivity [25]. The characterization results of NPs under different vacuum rotary evaporation times (1, 3, 6, and 10 h) are shown in Figure 3. The vacuum rotary evaporation enables the organic reagents in the rotating flask to diffuse and evaporate under negative pressure, greatly accelerating the evaporation process of organic reagents (Figure 3a). As shown in Figure 3b,c, after 1 h of vacuum rotary evaporation, the NPs had a larger particle size and a broader particle size distribution with a lower OVA adsorption efficiency (<60%) than others time points. In addition, the SEM images showed that the NPs exhibited poor dispersibility (Figure 3d). It may be due to the short duration of vacuum rotary evaporation and the incomplete removal of organic reagents, resulting in incomplete solidification of NPs. Therefore, many materials in the sample cannot form spherical structures, leading to lower OVA adsorption efficiency. After rotary evaporation for 3 h, the NPs exhibited an appropriate particle size and a narrow particle size distribution, with an OVA adsorption efficiency of approximately 75%. They demonstrated a poor spherical structure (Figure 3b,c,e). When rotary evaporation continued for 6 h, the NPs displayed a moderate particle size and distinctly spherical morphology (Figure 3b,f). It was worth noting that the NPs had the highest antigen adsorption efficiency at this time, which exceeded 80% (Figure 3c). However, when rotary evaporation is performed for 10 h, the NPs have a smaller particle size and higher antigen adsorption efficiency, but poorer uniformity (Figure 3b,c,g). It may be caused by a significant amount of water evaporated along with the organic solvent during long-term rotary evaporation, leading to poorer dispersibility of the NPs. After comprehensively considering factors, the vacuum rotary evaporation of 6 h was selected for further study.

#### 3.3.2. The Effect of Collection Methods of DDAB/PLA NPs on Physicochemical Characteristics of the Prepared NPs

**Figure 4:**
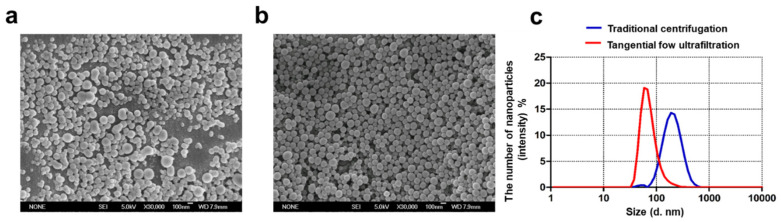
Characterization of DDAB/PLA NPs using different wash and collection methods in the scale-up preparation process. (**a**,**b**) SEM images of NPs using different wash and collection methods (×20,000). (**a**) Traditional centrifugation, (**b**) tangential flow ultrafiltration, and (**c**) particle size distributions of NPs.

To scale-up the process of DDAB/PLA NPs, it is necessary to apply other methods to replace the traditional centrifugation (TC) method for washing and collecting NPs. Tangential flow ultrafiltration (TFU) has been demonstrated as an efficient and green method for selecting the size and concentration of monodisperse NPs at various volume scales [26]. Therefore, this study aimed to compare the effects of TC and TFU methods on NPs, and the results are presented in Table 1. After washing and collection using the TC method, the particle size of the NPs was about 180 nm, and PDI was 0.154 ± 0.11, which was much higher than the particle size (150.3 ± 10.4) and PDI (0.090 ± 0.13) of the NPs prepared using the TFU method. SEM images also verified the advantages of NPs using the TFU method, in that the NPs had a relatively smooth and uniform surface (Figure 4a,b). In addition, from Figure 4c, the results displayed that NPs using the TFU method had a narrow particle size distribution. This meant the NPs prepared using the TFU method were more consistent in size, leading to more uniform physical and chemical properties, enhancing their performance and application effectiveness. Furthermore, a broad particle size distribution can decrease stability and increase the tendency for agglomeration or separation, thereby compromising their performance. During the process of washing and collecting NPs using the TC method, a significant loss of NPs occurred due to the limited centrifugation speed, which failed to separate and collect a large portion of small-sized NPs. Additionally, many NPs aggregated after high-speed centrifugation. Even with ultrasound assistance, achieving uniform dispersion was a challenge as excessive ultrasound power can affect the NPs’ shape and limit the antigen adsorption efficiency and yield. Hence, there was a low NP yield (<15%) from washing and concentration of NPs using the TC method. In contrast, the TFU method could effectively remove residual organic reagents. Moreover, this method showed a high yield (>90%) that was more beneficial for scaling up the production of NPs. Consequently, the TFU method was selected for the scale-up production of NPs.

### 3.4. Stability Evaluation of DDAB/PLA NPs

Long-term storage of NPs can lead to accumulation or precipitation, significantly reducing their dispersion and uniformity, which in turn impacts the bioavailability of the drugs and undermines their therapeutic potential [27,28]. Therefore, a robust evaluation of the stability of DDAB/PLA NPs is crucial. After a 6 h vacuum rotary evaporation process, the NPs were washed three times, concentrated using the TFU method, and assessed for stability at room temperature and 4 °C for 30 d. Samples from six groups of repeated experiments were characterized on days 0, 5, 10, 15, 21, and 30 during the observation period. 

**Figure 5:**
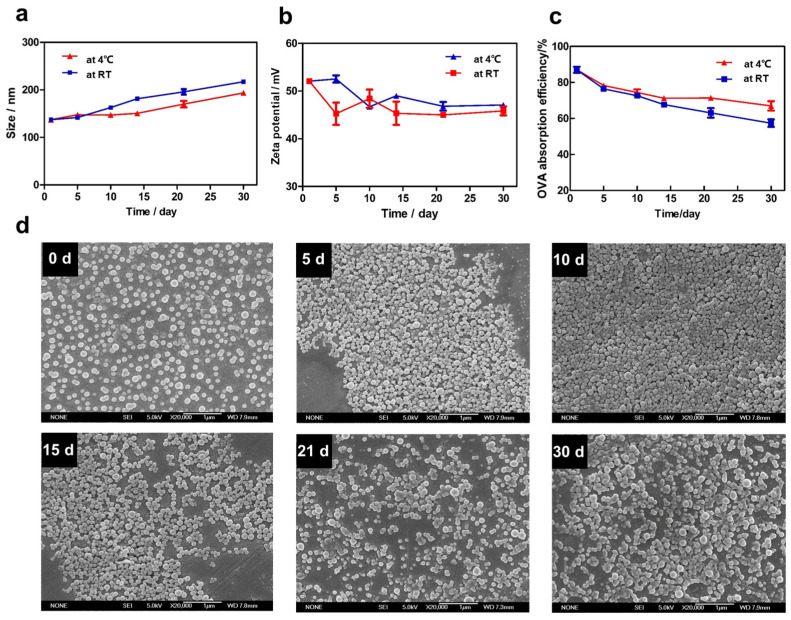
The stability evaluation of DDAB/PLA NPs: (**a**) particle size, (**b**) zeta potential, (**c**) antigen adsorption efficiency, and (**d**) representative SEM images of the NPs stored at 4 °C at different times 0, 5, 10, 15, 21, and 30 d (×20,000).

Supplementary Material Figure S1 shows that the NPs were uniformly dispersed in water, exhibiting a light blue milky appearance until 30 d. Although being stored at room temperature for 30 d, the appearance of the NPs remained unchanged. The characterization results are presented in Figure 5a–c; with increasing time, the size of NPs gradually increased, and the zeta potential slightly decreased. After 30 d, the NPs stored at room temperature had a particle size exceeding 200 nm, while those stored at 4 °C maintained a size of 130–150 nm within the first 15 d and slightly increased but remained below 200 nm after 30 d. The SEM images also confirmed that with prolonged storage time, the NPs gradually agglomerated, leading to an increase in particle size (Figure 5d). Figure 5c demonstrates that that on day 0, the NPs exhibited an antigen adsorption efficiency exceeding 80% for the model antigen OVA. However, as the storage time increased, the adsorption efficiency gradually decreased. After 30 d, the NPs stored at 4 °C maintained an antigen adsorption efficiency above 60%, while those stored at room temperature exhibited a faster decline, with an adsorption efficiency below 60%. This may be due to the decrease in the surface area of the agglomerated NPs, leading to a reduction of adsorption efficiency. In addition, the cationic lipid DDAB was modified on the PLA NPs through both embedding and adsorption mechanisms [29]. With prolonged storage time, some DDAB dissociated from the PLA NPs with a slight agglomeration between the NPs’ increased surface area, contributing to a decrease in antigen adsorption efficiency. To sum up, the DDAB/PLA NPs can be stored stably for 30 d at room temperature or 4 °C, with a preference for storage at 4 °C.

### 3.5. Detection of Organic Solvent Residues in DDAB/PLA NPs

**Figure 6:**
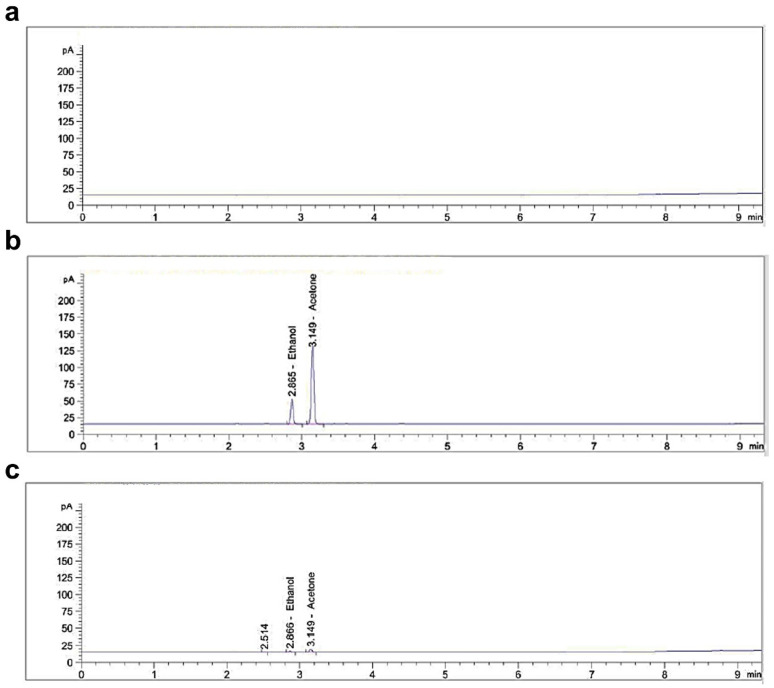
Identification and quantitative detection of organic residual solvents using gas chromatography (GC) method. Representative spectrum of (**a**) blank solvent (water), (**b**) reference solution, and (**c**) NP solution, respectively.

NPs use organic solvents to disperse and stabilize their components but may cause irritation and other side effects when injected into the body. Hence, conducting a thorough solvent residue analysis of NPs is essential to ensure their safety and stability for applications. The gas chromatography (GC) method was employed to identify and quantify residual solvents in this study. The blank solvent’s representative spectra are shown in Figure 6a, while the reference and NP solution are displayed in Figure 6b and c, respectively. It can be observed that the use of water as a blank solvent had no influence on the detection of ethanol and acetone under the chromatographic conditions, indicating reasonable method specificity. Figure 6 also revealed that the retention time of ethanol and acetone were 2.865 and 3.149 min, respectively. Both peaks exhibited efficiency separation (separation factor > 1.5) and no tailing phenomenon (asymmetric factor > 0.95). Hence, the GC conditions method could detect the residual concentrations of ethanol and acetone in the current study. The peak areas and average residual concentrations of ethanol and acetone in the NP solution are presented in Table 2 and Table S1. The residual concentration of ethanol and acetone in the 10 mg/mL NP sample was 0.02%, which met the requirements in Chinese Pharmacopoeia (Volume II, 2020 edition) [30] (not more than 0.5%). This result indicated that the DDAB/PLA NPs did not have side effects caused by residual solvents, which improved the safety and effectiveness of the vaccines.

### 3.6. Biosafety Evaluation of DDAB/PLA Nano-Vaccine

#### 3.6.1. Acute Toxicity Experiment in Mice

**Figure 7:**
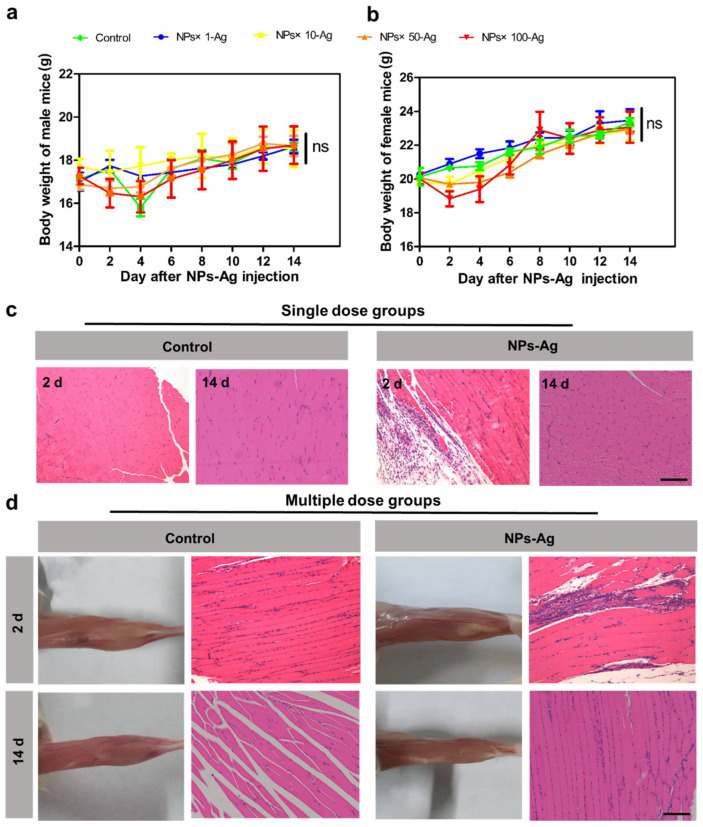
Evolution of the body weight of mice during acute toxicity testing: (**a**) male mice and (**b**) female mice (ns *p* < 0.05). (**c**) Results of muscle stimulation pathological section of the injection site in the single dose groups (H&E staining) and (**d**) multiple dose groups. bar = 200 μm.

Evaluating acute toxicity reactions and mortality rates after a single dosing in mice can reflect the degree of direct damage caused by vaccine preparations to the body, predict possible adverse reactions during clinical application, and provide a safe reference range for clinical practice. Acute toxicity experiments were conducted to evaluate the vaccine’s safety, and the weight changes of mice in each group during the observation period are shown in Figure 7a,b. The results demonstrated that the mice exhibited good DDAB/PLA nano-vaccine tolerance. No deaths occurred even at a dose 100 times higher (30 mg/mouse) than the normal immunization dose. During the study period, it was evident that there was an increase in the mean group body weight in all groups. There was no statistically significant difference in the mean group body weight gain at the end of the study period between the treated and the control groups. Food consumption was within the normal range in both sexes during the study period. In addition, the mice displayed good mental status, normal food intake, fur color, bowel movements, and urination. Therefore, at the examined dose levels, the nano-adjuvant vaccine showed no observable toxicity in mice, and the Maximum Tolerated Dose (MTD) was determined to be greater than 30 mg/mouse.

#### 3.6.2. Evaluation of Inflammation at the Injection Site after Single and Multiple Immunizations

The vaccine formulations can effectively recruit antigen-presenting cells and inflammatory cells at the injection site, thus potentiating the generation of immune responses within the body [31,32]. It is essential to evaluate the stimulation of muscle tissue by the DDAB/PLA NPs vaccine to determine whether the injection of the novel vaccine formulation would lead to severe and irreversible inflammatory reactions at the injection site. As shown in Figure 7c, the results obtained 2 and 14 d after single administration indicated that the mice exhibited regular activity, food intake, and water consumption. No severe erythema or ulceration was observed at the injection site. Compared to the control group, the mice in the NPs-Ag group exhibited mild inflammation in the muscle tissue at the injection site 2 d after a single administration. However, this inflammation usually resolves 14 days later. In the multiple-dose group, evident lymphocytic infiltration and slight morphological changes were observed in focal skeletal muscle cells at the injection site after 2 d, which resolved after a recovery period of 14 d, with the inflammation being largely eliminated (Figure 7d). This may be because, during the early stages of vaccine injection, the lymphocytes were recruited to the injection site, promoting the production of protective immune responses [33]. However, as the recovery period progresses, the inflammation gradually resolves, and the nodules formed at the injection site decrease, indicating that the vaccine formulation did not significantly stimulate the muscle tissue. Therefore, the DDAB/PLA NPs can enable slow antigen release and possess biodegradable characteristics, making them a suitable candidate for vaccine delivery systems. With further research and development, it could potentially improve vaccine efficacy and provide more sustained immunological responses.

#### 3.6.3. Organ Weight and Microscopic Histopathological Changes in Repeated Toxicity Study

**Figure 8:**
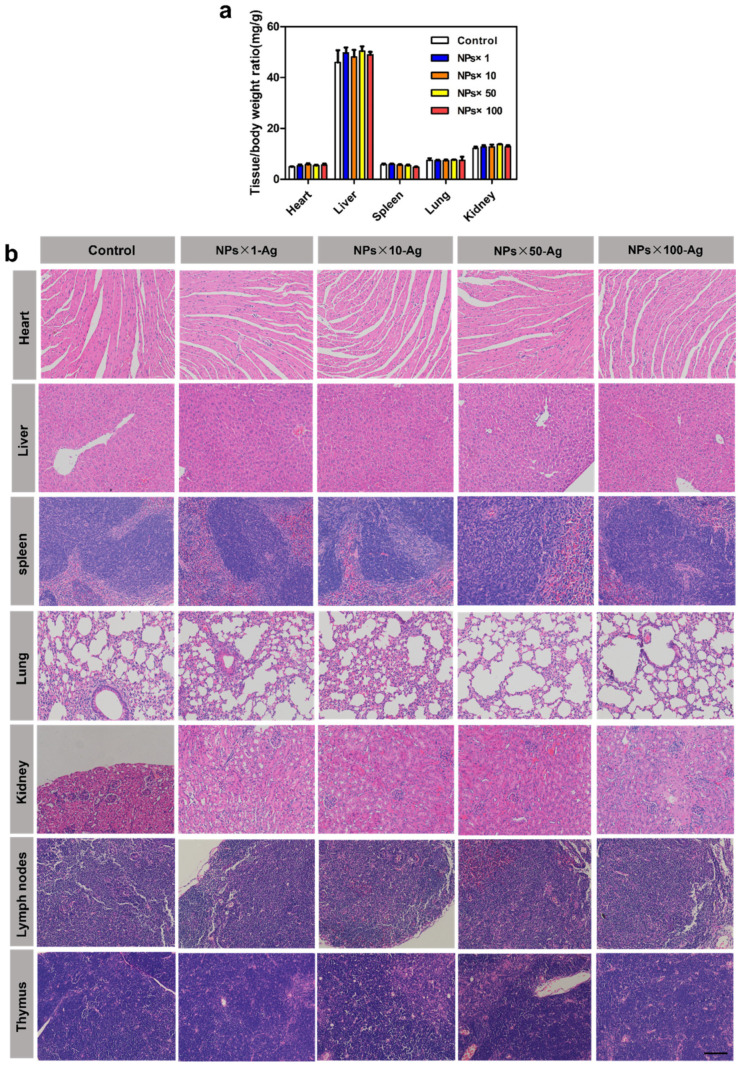
Results of the repeated dose toxicity test. (**a**) Major organs to body weight ratio for each group of mice; (**b**) H&E staining of major tissues, including heart, liver, spleen, lung, kidney, lymph nodes, and thymus (bar = 100 μm).

When the animal’s body undergoes pathological changes, it can be reflected in altered weights of the affected organs, leading to changes in organ coefficients. An increase in organ coefficient indicates the possible presence of issues such as congestion, edema, or hypertrophy in the organ. At the same time, a decrease may suggest corresponding organ atrophy and other degenerative changes [34]. In this study, a standard immunization procedure was simulated by immunizing mice with the vaccine formulation containing the DDAB/PLA NPs at concentrations of 10, 50, and 100 times the regular dose and observing continuously for 30 d. On day 30, the weights of major organs in each group of mice were tested, and the results are presented in Table S2, while the organ coefficients of significant organs are shown in Table S3 and Figure 8a.

It can be concluded that there were no significant changes in the weights of the major organs in mice at the end of the study, and no significant differences were observed in organ coefficients among the groups. Furthermore, the histological sections stained with H&E of significant organs, thymus, and draining lymph nodes from each group of mice after fixation are shown in Figure 8b. The cardiac myocardial tissue of all mouse groups exhibited intact structure, with orderly myocardial fiber arrangement and normal myocardial cell morphology, without any evident vascular congestion or inflammatory cell infiltration. Upon examination of liver tissue, no significant difference was observed between the normal adjuvant dose group (NPs × 1-Ag) and the higher adjuvant dose group (NPs × 10-Ag) compared to the control group. However, a slightly increased number of infiltrating inflammatory cells were observed in the high adjuvant dose group (NPs × 50-Ag) and the high adjuvant dose group (NPs × 100-Ag). As the liver is the primary detoxification organ, a few infiltrating inflammatory cells are considered within the normal range. Moreover, all mouse groups displayed a small number of vacuoles, which might be associated with the mice’s diet and require further investigation. The spleen exhibited distinct red and white pulp boundaries without significant abnormalities. In addition, lung analysis revealed clear and intact reticular structures without any obvious abnormality in all mouse groups compared to the control group. The kidney results indicated that the glomerular structure was intact, with no apparent dilation or shrinkage of renal tubules and no significant abnormality in the renal cortex structure. Examination of the draining lymph node showed that the follicular structure in all mouse groups was intact without any obvious abnormality. Analysis of the thymus revealed an average cortical thickness without apparent atrophy in all mouse groups.

The initial manifestation of a cytokine storm is primarily characterized by high fever [35]. Hence, the rectal temperature of mice in each group was measured before injection (t0) and 4 h after injection (t4) of the respective formulations, and the results are summarized in Table S4. It was observed that the body temperature of the mice slightly rose after the injection of the vaccine formulations in all groups at 4 h post-injection, compared to their pre-injection temperatures. Nevertheless, these temperatures remained within the normal physiological range. Furthermore, even during the repeated toxicity study, when the nano-adjuvant dose was 100 times the regular dose, the vaccine formulation did not cause significant organ damage or trigger an abnormal fever in the mice.

## 4. Conclusions

The study successfully developed the DDAB/PLA NPs using the nanoprecipitation method, achieving uniform particle size distribution and efficient antigen loading. Additionally, the process parameters for scale-up production were optimized to facilitate large-scale production. In acute toxicity tests, even at a 100-fold increase in adjuvant dose, no deaths or adverse behaviors were observed in the mice, indicating good tolerance of the vaccine formulation. The local irritation experiment showed no necrosis or pathological changes at the injection site following single or multiple injections of the vaccine formulation at the normal immunization dose. This suggested the reversibility of muscular irritation caused by the nano-adjuvant vaccine in mice. The repeated toxicity study using H&E staining further confirmed the safety of the vaccine formulation in the mice, revealing no significant necrosis or severe lesions in their organs. Furthermore, no abnormal increase in body temperature was observed after injection, ruling out the occurrence of dangerous conditions like cytokine storms. A comprehensive biosafety evaluation indicated that the DDAB/PLA NPs had the potential for clinical translation as a novel vaccine adjuvant. The results provided valuable insights for developing effective and safe vaccine adjuvants in the future.

## Figures and Tables

**Table 1:** Characterization of DDAB/PLA NPs was prepared using different wash and collection methods.

	Particle Size (nm)	PDI	Zeta Potential (mV)	Adsorption Efficiency (%)	Yield (%)
TC	180.9 ± 7.8	0.154 ± 0.11	47.2 ± 2.5	82.5 ± 1.5	11.8 ± 3.6
TFU	150.3 ± 10.4	0.090 ± 0.13	54.0 ± 4.0	85.4 ± 4.0	96.3 ± 5.2

TC is traditional centrifugation; TFU is tangential flow ultrafiltration.

**Table 2:** The results of mean peak area, RSD, and solvent concentration of ethanol and acetone using the GC method.

Group	Solvent Type	Mean Peak Area ± SD	RSD (%)	Solvent Concentration (mg/mL)
Reference solution	Ethanol	89.1376 ± 12.37	2.64	0.05
	Acetone	313.28 ± 4.56	1.46	0.05
NPs solution	Ethanol	3.96 ± 0.12	3.03	0.02
	Acetone	13.03 ± 0.24	1.84	0.02

## Data Availability

The datasets used and analyzed during the current study are available from the corresponding author upon request.

## Supplementary Materials

The following supporting information can be downloaded at: https://www.mdpi.com/article/10.3390/jfb15050127/s1, Figure S1: Pictures of DDAB/PLA NPs stored for 30 d, (a) Storage at room temperature and (b) Storage at 4 °C; Table S1: Peak areas of the reference solution and DDAB/PLA NPs by the GC method; Table S2: Main organ weight of each group of mice in repeated toxicity study; Table S3: Major organ coefficient of each group of mice in repeated toxicity study; Table S4: Mouse rectal temperature.
