# Supplementary material for: Process Optimization of Scaled-Up Production and Biosafety Evaluation of the Dimethyl-Dioctadecyl-Ammonium Bromide/Poly(lactic acid) Nano-Vaccine
Source: J Funct Biomater. 2024 May 14;15(5):127. doi: 10.3390/jfb15050127 (PMC11122170; doi:10.3390/jfb15050127)
Supplement: Supplementary file 1 [file jfb-15-00127-s001.zip › jfb-2964762-supplementary.pdf]

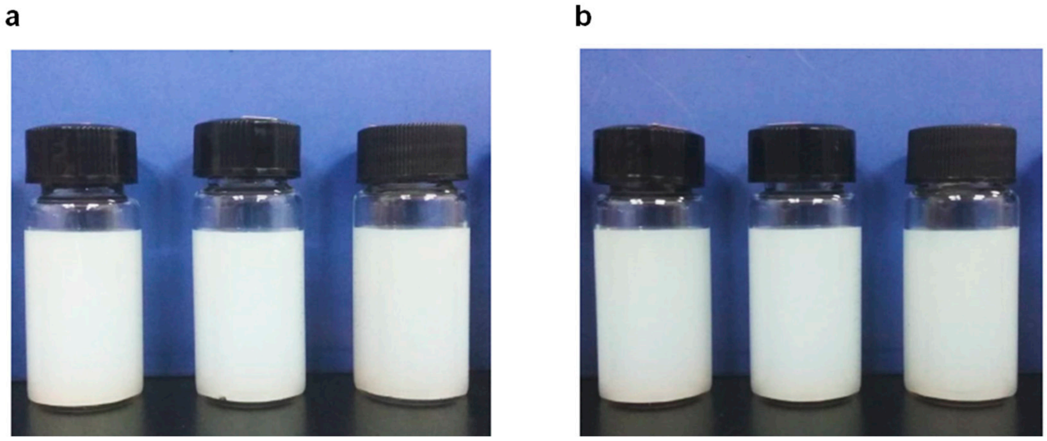

Figure S1. Pictures of DDAB/PLA NPs stored for 30 d. (a) Storage at room temperature and (b) Storage at 4℃.

Table S1 Peak areas of the reference solution and DDAB/PLA NPs by the GC method.

Group	Solvent type	Peak area					
Reference solution	Ethanol	4.12	3.96	3.89	4.08	3.83	3.86
	Acetone	12.81	12.74	13.36	12.95	13.08	13.22
NPs solution	Ethanol	87.67	89.54	91.59	87.09	89.32	93.36
	Acetone	304.19	314.86	315.69	314.23	314.02	316.71

Table S2 Main organ weight of each group of mice in repeated toxicity study.

Group	Organ weight / g				
	Heart	Liver	Spleen	Lung	Kidney
Control	0.13±0.03	1.31±0.22	0.13±0.03	0.17±0.02	0.29±0.04
NPs×1-Ag	0.11±0.02	1.04±0.08	0.12±0.01	0.15±0.01	0.27±0.02
NPs×10-Ag	0.10±0.02	0.93±0.01	0.1±0.01	0.14±0.02	0.25±0.01

NPs×50-Ag	0.12±0.01	1.05±0.07	0.1±0.03	0.14±0.01	0.28±0.01
NPs×100-Ag	0.12±0.01	1.05±0.11	0.1±0.01	0.42±0.45	0.29±0.02

Table S3 Major organ coefficient of each group of mice in repeated toxicity study.

Group	Organ weight/g				
	Heart	Liver	Spleen	Lung	Kidney
Control	4.39±0.85	43.24±7.38	4.41±1.13	5.54±0.81	9.66±1.16
NPs×1-Ag	5.69±1.11	45.98±6.23	5.5±1.21	7.53±0.74	13.31±0.6
NPs×10-Ag	4.58±0.92	44.47±0.57	4.6±0.43	6.91±0.75	11.73±0.35
NPs×50-Ag	5.54±0.65	49.86±3.31	4.74±1.3	6.62±0.61	13.44±0.46
NPs×100-Ag	5.83±0.38	50.13±5.14	4.92±0.1	7.37±1.94	14.04±1.04

Table S4 Mouse rectal temperature.

Group	0 d		14 d		28 d	
	t0	t4	t0	t4	t0	t4
Control	36.80±0.20	36.87±0.15	36.67±0.23	36.80±0.35	36.50±0.17	36.77±0.21
NPs×1-Ag	36.60±0.06	36.93±0.45	36.67±0.35	37.03±0.29	36.73±0.32	36.83±0.32
NPs×10-Ag	36.33±0.10	36.70±0.10	36.43±0.20	36.00±0.40	36.60±0.58	37.00±0.17
NPs×50-Ag	36.50±0.38	36.70±0.10	36.43±0.21	37.20±0.50	36.73±0.25	37.10±0.20
NPs×100-Ag	36.83±0.46	37.50±0.36	36.70±0.10	37.50±0.36	36.70±0.36	37.53±0.15
